# Knowledge and Awareness of Medication-Related Osteonecrosis of the Jaw Among Dental Practitioners in Mumbai: A Questionnaire-Based Survey

**DOI:** 10.7759/cureus.76448

**Published:** 2024-12-26

**Authors:** Murtaza A Shapurwala, Viraj Kharkar, Saudamini More, Harjit Kalsi, Sanpreet S Sachdev

**Affiliations:** 1 Oral and Maxillofacial Surgery, Bharati Vidyapeeth (Deemed to be University) Dental College and Hospital, Navi Mumbai, IND; 2 Public Health Dentistry, Bharati Vidyapeeth (Deemed to be University) Dental College and Hospital, Navi Mumbai, IND; 3 Oral Pathology and Microbiology, Bharati Vidyapeeth (Deemed to be University) Dental College and Hospital, Navi Mumbai, IND

**Keywords:** antiangiogenic drug, bisphosphonates, chemotherapy, oncology, osteonecrosis

## Abstract

Introduction: Osteonecrosis of the jaw resulting from the adverse effects of medical treatments is known as medication-related osteonecrosis of the jaws (MRONJ). The knowledge of dental professionals about this condition is crucial in addressing it on a regional and global scale. This study aims to assess the knowledge and awareness of MRONJ among dental practitioners in Mumbai.

Materials and methods: This descriptive cross-sectional study utilized a web-based questionnaire comprising 21 questions distributed to dental professionals and postgraduate students at dental colleges, hospitals, and private practices in Mumbai.

Results: A total of 403 dentists participated in the survey, with 85% familiar with the condition. A significantly (p < 0.05) higher proportion of clinicians with over 20 years of experience and those specializing in oral and maxillofacial surgery were aware of the indications for bisphosphonate drugs. About 53% of oral surgeons and 57.7% of participants with more than 20 years of experience were aware that antiangiogenic drugs are used in breast cancer treatment. However, awareness of their use in lung cancer was only about 21%.

Conclusions: Although practitioners were familiar with the term MRONJ, their knowledge of the drugs affecting the disease, as well as its clinical and radiographic presentations, was inadequate. Awareness of treatment strategies and recent advances was particularly lacking among general dentists and other specialists apart from oral and maxillofacial surgeons (OMFS). Practitioners also identified a lack of an interdisciplinary approach. There is a clear need for enhanced education and awareness on this subject, which can be achieved by conducting interdisciplinary surveys, conferences, and seminars.

## Introduction

“Osteonecrosis” refers to the death of bone tissue, which can occur due to various etiological factors, including traumatic injuries, cytotoxicity, bone marrow depression, radiation therapy, excessive alcohol consumption, or certain medical treatments [1]. The direct cytotoxic effects of drugs such as bisphosphonates, antiresorptives, and antiangiogenics on osteocytes/osteoblasts contribute to this condition, particularly in patients undergoing these treatments. This type of osteonecrosis resulting from the adverse effects of medical treatments that primarily affect the jawbones is termed medication-related osteonecrosis of the jaws (MRONJ) [2,3]. 

The term MRONJ, coined by the American Association of Oral and Maxillofacial Surgery (AAOMS), refers to the exposed bone caused by drugs that fail to heal for at least eight weeks [4]. Its prevalence varies from 0.001% to 0.2% across different countries, with a direct correlation to the duration of therapy and drug dosage [5-7]. MRONJ can cause tremendous pain, infections, and even pathological fractures of the jawbones, significantly impairing the patient’s ability to perform masticatory and speech functions, thus negatively impacting their quality of life [8].

Currently, no treatments effectively address MRONJ. As a result, the primary objective for optimal patient care is prevention and palliative management [9]. The knowledge of dental professionals about this condition is crucial in addressing it on a regional and global scale. However, recent cross-sectional studies have shown that medical and dental practitioners, as well as students, are largely unaware of various aspects of MRONJ [10-13].

The majority of dental practitioners in India are concentrated in urban areas, with less than 5% practicing in rural areas. This makes urban areas more suitable for studying the attitudes of dental practitioners, with more generalizable results. Recently, e-questionnaires have gained popularity due to their feasibility, quick response time, and ability to record comprehensive data. Thus, the present questionnaire-based study was conducted to assess the knowledge and awareness of MRONJ among dental practitioners in Mumbai. This study aims to evaluate the understanding of the diagnostic criteria and risk factors for MRONJ among dental practitioners in Mumbai.

## Materials and methods

Study design

This cross-sectional study was conducted in Bharati Vidyapeeth Dental College and Hospital, Terna Dental College, DY Patil School of Dentistry, YMT Dental College and Hospital, and MGM Dental College and Hospital in Navi Mumbai, from May to October 2022. The Ethics Committee of the institute approved the study (document number: IEC341072022). A structured Google Forms questionnaire was distributed to dental faculties, postgraduate students working in the five dental colleges, and dental professionals in Mumbai. The questionnaire consisted of two parts: part I contained basic information about the practitioners' age, years of experience, and specialization, while part II assessed their knowledge of MRONJ, its treatment, and recent advances. Questions validated from earlier studies, with certain modifications, were included.

Sampling and validation

The sample size was calculated assuming a 50% knowledge level in the population and a 5% type 1 error. Based on these parameters, a sample size of n = 403 participants was deemed adequate for the study. The participants comprised dentists from various specialties and sectors across Mumbai, including general dentists, dental specialists, and postgraduate students. Undergraduate students and interns were excluded. The sample was obtained using a convenience sampling technique. The questionnaire was face-validated by experts, assessing criteria such as ease of comprehension, linguistic quality, repetitiveness, appropriateness for the objective, and incorporating constructive feedback. Each question received a content validity index score of 0.80 or higher. Minor grammatical and syntactical errors were identified during internal validation. The questionnaire was pilot-tested on a homogenous group of 20 practitioners, and the feedback received was satisfactory.

Data collection

The questionnaires were sent electronically via messages and emails to the target population, accompanied by a cover letter briefly describing the study. Informed consent was obtained from all study participants, with assurance that their participation was voluntary and their confidentiality would be maintained.

Data analysis

Data were entered into Microsoft Excel (Microsoft Corp., Redmond, WA, United States) and analyzed using IBM SPSS Statistics for Windows, Version 20 (Released 2011; IBM Corp., Armonk, NY, United States). Descriptive statistics were calculated as frequency and percentage. For subgroup analysis, the participants were categorized by specialty (general dentists, oral and maxillofacial surgeons (OMFS), and other specialties) and by years of experience (<1 year, 1-20 years, >20 years). The chi-square test was used to compare various subgroups based on independent variables such as age, gender, qualification, years of experience, and dental specialty. A p < 0.05 was considered statistically significant.

## Results

A total of 403 dental students, faculty, and practitioners participated in the survey, with 40 participants having less than one year of experience, 337 with 1-20 years of experience, and 26 participants with more than 20 years of experience. About 85% (n = 342) of the participants were familiar with the term MRONJ. Regarding the source of their knowledge, 29% (n = 117) gained knowledge from journals and literature, 22% (n = 89) from their undergraduate program, 19.8% (n = 80) from their postgraduate training, and the remaining participants from seminars, the Internet, drug experiences, and patient anamnesis. About 92% (n = 371) recognized the importance of a dental examination before starting bisphosphonate therapy, and 84.9% (n = 341) acknowledged the need for oral cavity prophylaxis before initiating bisphosphonate therapy. Moreover, 57% (n = 230) knew that bisphosphonates are administered both intravenously and orally. However, only 30.5% (n = 123) correctly identified the drugs responsible for the risk of developing MRONJ.

It was observed that a significantly (p < 0.05) higher number of clinicians with more than 20 years of experience and those specializing in oral and maxillofacial surgery were aware of the indications for bisphosphonate drugs (Table 1). About 51.72% (n = 30) of the OMFS and 57.69% (n = 15) of the participants with more than 20 years of experience were aware that antiangiogenic drugs are used for breast cancer. However, awareness of their use in lung cancer was only about 11.53% (n = 3) (Table 2).

**Table 1:** Comparison of awareness regarding the indications for bisphosphonates based on dental specialization and clinical experience among the study participants. Test applied: chi-square test. p < 0.05 is statistically significant.

Conditions treated with bisphosphonates	Dental specialization			Chi-square	p-value	Clinical experience			Chi-square	p-value
	General dentists (n = 27)	Oral and maxillofacial surgeons (n = 58)	Other dental specialties (n = 318)			<1 year (n = 40)	1-20 years (n = 337)	>20 years (n = 26)		
Osteoporosis	74.07% (20)	84.48% (49)	78.93% (251)	1.506	0.471	60.00% (24)	81.89% (276)	88.46% (23)	10.218	0.006
Multiple myeloma	11.11% (3)	50% (29)	38.36% (122)	11.4	0.03	5.00% (2)	39.16% (132)	57.69% (15)	12.442	0.002
Bone metastasis	18.51% (5)	58.62% (34)	30.18% (96)	19.609	0	15.00% (6)	34.12% (115)	50.00% (13)	8.618	0.013
Anemia	0.00% (0)	0.00% (0)	0.31% (1)	0.272	0.873	0.00% (0)	0.29% (1)	0.00% (0)	0.19	0.909
Osteoporosis imperfecta	14.81% (4)	31.03% (18)	21.06% (67)	3.428	0.18	27.50% (11)	19.58% (66)	42.30% (11)	7.619	0.022
Paget's disease	48.14% (13)	75.86% (44)	45.97% (160)	14.027	0.001	52.50% (21)	53.70% (181)	53.84% (14)	0.02	0.99
Hypertension	0.00% (0)	0.00% (0)	0.62% (2)	0.545	0.761	0.00% (0)	0.59% (2)	0.00% (0)	0.381	0.827
I don't know	7.40% (2)	0.00% (0)	5.03% (16)	3.485	0.175	10.00% (4)	3.56% (12)	3.84% (1)	4.115	0.128

**Table 2:** Comparison of knowledge regarding the indication of antiangiogenic drugs depending on dental specialization and clinical experience among the study participants. Test applied: chi-square test. p < 0.05 is statistically significant.

Conditions treated with antiangiogenic drugs	Dental specialization			Chi-square	p-value	Clinical experience			Chi-square	p-value
	General dentists (n = 27)	Oral and maxillofacial surgeons (n = 58)	Other dental specialties (n = 318)			<1 year (n = 40)	1-20 years (n = 337)	>20 years (n = 26)		
Prostate cancer	29.62% (8)	50% (29)	28.61% (91)	10.41	0.005	27.50% (11)	31.75% (107)	26.92% (7)	0.162	0.922
Breast cancer	37.03% (10)	51.72% (30)	50.31% (160)	2.54	0.31	42.50% (17)	51.63% (174)	57.69% (15)	1.721	0.423
Multiple myeloma	3.70% (1)	50% (29)	40.25% (128)	17.26	0	25.50% (9)	40.65% (137)	42.30% (11)	4.244	0.12
Osteoporosis	11.11% (3)	13.79% (8)	11.94% (38)	0.186	0.911	20.00% (8)	10.97% (37)	3.84% (1)	4.52	0.104
Bone metastasis	3.70% (1)	36.20% (21)	29.24% (93)	10	0.007	12.5% (5)	31.45% (106)	23.07% (6)	6.165	0.046
Paget's disease	3.70% (1)	31.03% (18)	19.81% (63)	8.759	0.013	12.50% (5)	21.66% (73)	15.38% (4)	1.929	0.381
Malign hypercalcemia	3.70% (1)	34.48% (20)	16.66% (53)	14.535	0.001	7.50% (3)	18.69% (63)	30.76% (8)	5.454	0.065
Lung cancer	14.81% (4)	34.48% (20)	19.98% (54)	9.615	0.008	15.00% (6)	20.47% (69)	11.53% (3)	1.59	0.452
I don't know	59.25% (16)	13.79% (8)	27.98% (89)	18.877	0	45.00% (18)	26.11% (88)	26.92% (7)	5.596	0.051

**Figure 1:**
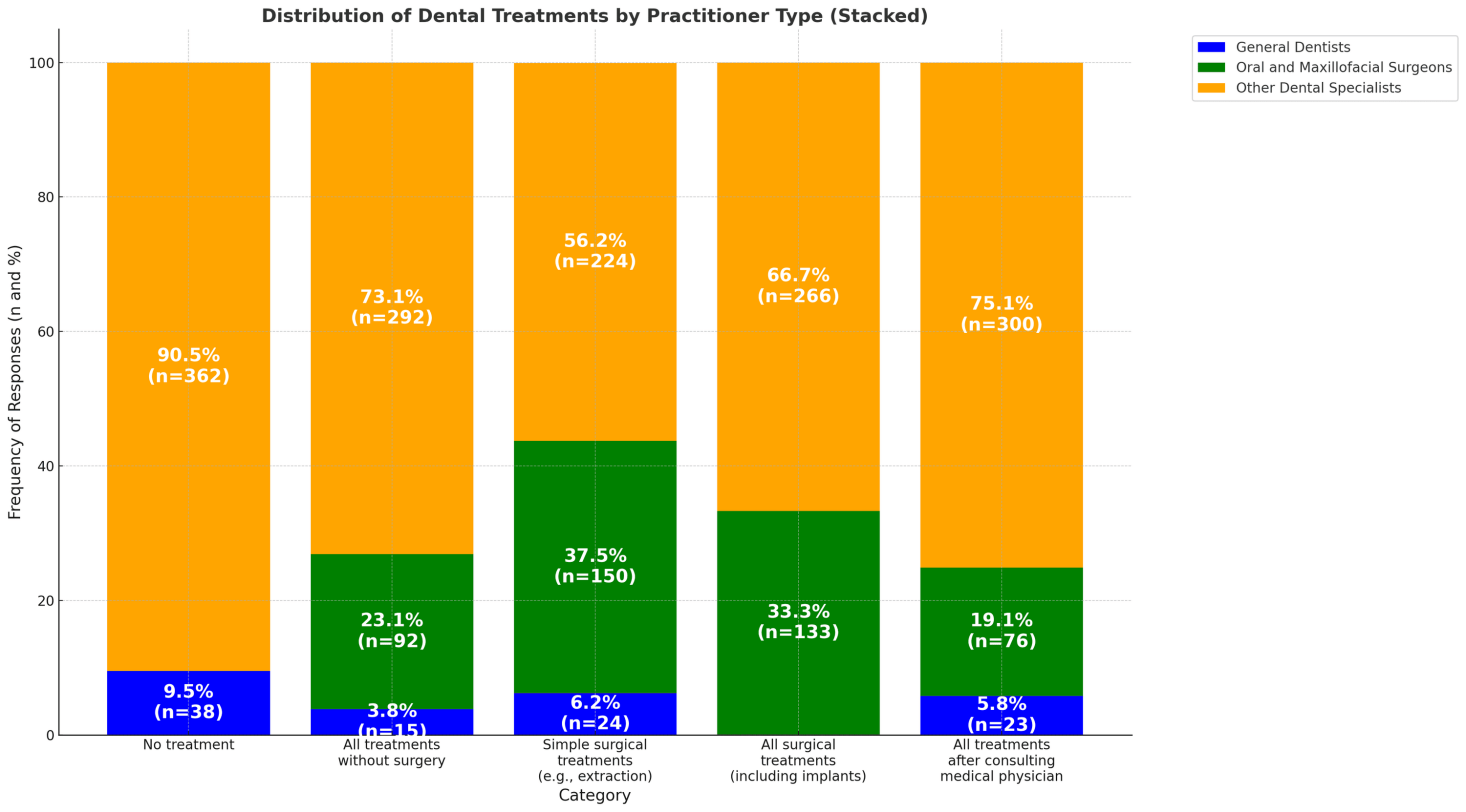
Knowledge of MRONJ treatment among the study participants.

Similarly, the OMFS and participants with more than 20 years of experience were significantly more familiar with the clinical and radiographic features of MRONJ (Table 3). They also exhibited significantly higher awareness of regenerative materials and teriparatide, particularly among practitioners with more than 20 years of experience (p < 0.05) (Table 4). Furthermore, overall awareness was significantly higher in the OMFS group compared to others, as shown in Table 4. According to Figure 1, the participants selected their treatment strategies. Interestingly, 90% (n = 362) of individuals in other specialties decided not to perform any treatment. In contrast, only 19.1% (n = 76) of the OMFS opted for an interdisciplinary approach. 

**Table 3:** Comparison of knowledge of clinical and radiographic findings of MRONJ depending on dental specialization and clinical experience among the study participants. Test applied: chi-square test. p < 0.05 is statistically significant.

Clinical and radiographical findings of MRONJ	Dental specialization			Chi-square	p-value	Clinical experience			Chi-square	p-value
	General dentists (n = 27)	Oral and maxillofacial surgeons (n = 58)	Other dental specialties (n = 318)			<1 year (n = 40)	1-20 years (n = 337)	>20 years (n = 26)		
Halitosis	40.74% (11)	75.86% (44)	40.88% (130)	24.096	0	30.00% (12)	47.77% (161)	50.00% (13)	5.106	0.078
Ulceration of the oral mucosa	33.33% (9)	72.41% (42)	49.37% (157)	14.851	0.001	22.50% (9)	52.81% (178)	76.92% (20)	18.783	0
Drainage	22.22% (6)	56.89% (33)	35.84% (114)	12.617	0	27.50% (11)	39.16% (132)	34.61% (9)	2.741	0.257
Mobile and sensitive teeth	40.74% (11)	46.55% (27)	41.19% (131)	13.648	0.001	32.50% (13)	52.81% (178)	57.69% (15)	6.853	0.032
Exposed bone region	14.81% (4)	31.03% (18)	21.06% (67)	0.637	0.727	40.00% (16)	41.24% (139)	53.84% (14)	1.643	0.44
Thickening of the lamina dura	11.11% (3)	5.17% (3)	17.61% (56)	6.383	0.041	15.00% (6)	15.13% (51)	15.38% (4)	0.004	0.998
Enlargement of the periodontal space	22.22% (6)	29.31% (17)	20.12% (64)	10.512	0.005	10.00% (4)	24.62% (83)	23.07% (6)	3.781	0.151
Structure change of the bone trabecula	55.55% (15)	55.17% (32)	56.91% (181)	0.075	0.963	50.00% (20)	57.86% (195)	50.00% (13)	1.506	0.471
Sequestration area	51.85% (14)	62.06% (36)	65.09% (207)	2.692	0.26	37.50% (15)	66.76% (225)	65.38% (17)	13.377	0.001
I don't know	22.22% (6)	5.17% (3)	16.03% (51)	5.626	0.06	32.50% (13)	14.83% (50)	11.53% (3)	9.616	0.008

**Table 4:** Comparison of MRONJ awareness based on years of clinical experience and dental specialization among the study participants. Test applied: chi-square test. p < 0.05 is statistically significant.

Awareness questions	Yes/no	Years of experience			p-value	Chi-square	Dental specialization			p-value	Chi-square
		<1 year	1-20 years	>20 years			General dentists	Oral and maxillofacial surgeons	Other dental specialties		
		(n = 40)	(n = 337)	(n = 26)			(n = 27)	(n = 58)	(n = 318)		
Drug holiday concept	Yes	7	108	10	0.19	4.256	3	46	76	0	75.75
		17.50%	32%	38.40%			11.10%	79.30%	23.80%		
	No	33	229	16			24	12	242		
		82.50%	68%	61.60%			88.90%	20.70%	76.20%		
Stage-specific treatment strategies for MRONJ	Yes	7	106	11	0.082	5	3	4	242	0	66.6
		17.50%	31.40%	42.30%			11.10%	75.80%	24.20%		
	No	28	231	15			24	14	241		
		82.50%	68.60%	57.70%			88.90%	24.20%	75.80%		
Use of regenerative material during the surgical treatment of MRONJ	Yes	5	105	9	0.042	6.327	4	33	82	0	25.82
		12.50%	31.10%	34.60%			14.80%	56.80%	25.70%		
	No	35	232	17			23	25	236		
		87.50%	68.90%	65.40%			85.20%	43.20%	74.30%		
Use of teriparatide for the treatment of MORNJ	Yes	1	58	7	0.019	7.907	0	21	45	0	23.092
		2.50%	17.20%	26.90%			0%	36.30%	14.20%		
	No	39	279	19			27	37	273		
		97.50%	82.80%	73.10%			100%	63.70%	85.80%		

## Discussion

The present study aimed to assess the understanding of MRONJ among dental practitioners and faculty in Mumbai, India's financial capital, with a population of more than 21.6 million. Cancer prevalence in India is notable, with lung and breast cancers being the most common, affecting nearly two million individuals [6]. Over 90% of MRONJ cases are observed in cancer patients with bone metastases receiving high doses of medication [14]. As the elderly population with osteoporosis and cancer-related conditions grows, the use of antiresorptive and antiangiogenic drugs has become increasingly important [15]. Bisphosphonates, commonly used to treat conditions such as osteoporosis, multiple myeloma, Paget's disease, bone metastases, and osteogenesis imperfecta, can be administered orally or intravenously, with the intravenous route providing a more rapid effect [2]. In this study, while only 57% of participants (n = 230) were aware of both administration routes for bisphosphonates, OMFS exhibited greater awareness compared to general dentists or those from other dental specialties. This may be due to the higher frequency of oncology patients undergoing radiation therapy, which is associated with MRONJ. Additionally, a positive correlation was observed between the professionals' experience and their awareness of MRONJ, emphasizing the role of experience in diagnosing such rare conditions. 

It is worth noting that antiangiogenic drugs, commonly associated with MRONJ, are typically prescribed for the treatment of breast, prostate, colorectal, non-small cell lung, and renal cell carcinomas and bone metastasis [3]. These drugs work by inhibiting vascular endothelial growth factors (VEGFs) to prevent cancer spread through the blood and lymphatic systems, but they can also unintentionally contribute to osteonecrosis [16]. The present study found a general lack of knowledge about antiangiogenic drugs, particularly among participants with less than one year of experience in the field. OMFS were more likely to identify halitosis, mucosal ulceration, and sequestration areas as the most common signs of MRONJ, while general dentists and other specialists were more likely to note changes in bone trabecular structure and areas of bony sequestration.

To assess the clinical and radiographical factors of MRONJ, AAOMS has developed a staging system [12]. The first noticeable change in X-rays is the thickening of the lamina dura, an aspect that was surprisingly underrecognized in the present study. Additionally, the system emphasizes the reduction in the periodontal ligament space as a key indicator. On the contrary, the majority of the participants believed that the widening of the periodontal ligament was associated with periodontitis, which negates the actual pathogenesis of MRONJ. Identifying mobility, exposed bone regions, and inflammation as indications of periodontal disease is important for identifying undiagnosed MRONJ in patients. Although halitosis was a common response among the majority of the participants, it is not so commonly observed in MRONJ cases. A recent study on approximately 200 patients with osteoradionecrosis of the jaws reported halitosis in only 1% of cases [17]. Therefore, it is crucial to adhere to the AAOMS staging guidelines and be aware that halitosis is not a typical symptom of MRONJ.

Studies have shown that preoperative drug discontinuation significantly reduces the incidence of MRONJ [18]. According to Patil et al., across multiple centers, only 31.6% of dentists were familiar with the concept of a drug holiday [11]. In the present study, OMFS demonstrated relatively higher awareness, though overall awareness was still low. A similar trend of awareness was observed regarding the stage-specific treatment strategies for MRONJ, with OMFS and participants with more than 20 years of experience showing greater knowledge. In a study by Al-Eid et al. among dentists in Saudi Arabia, an even lower awareness regarding the prevention and management of MRONJ was observed (3%) [12].

Commonly used therapeutic measures for MRONJ include bone morphogenetic proteins and autologous platelet concentrates. Teriparatide, which mimics human parathyroid hormone and promotes bone growth at low doses, has also shown potential as a promising treatment for MRONJ [19]. OMFS and those with more than 20 years of experience had a deeper comprehension of stage-specific treatment compared to general dentists and other specialists, who had lower awareness levels.

It is crucial to emphasize that prevention is more important than the management of MRONJ, which can be efficiently achieved through collaboration between oncologists and dentists. Patients at increased cancer risk should be educated on the significance of oral hygiene and regular dental check-ups [20]. A concerning finding in the present study was that tooth extractions and implants are frequently recommended surgical procedures for MRONJ patients by OMFS and other specialists. Overall, a multidisciplinary management approach that adheres to the stage-specific treatment prescribed by AAOMS is recommended [14].

A limitation of this study was the selection and volunteer biases introduced by convenience sampling, particularly among OMFS and other dental specialists. Nonresponse bias and survey fatigue also affected participation, with fewer general practitioners taking part. Older, more experienced practitioners, who may be less familiar with technology, were less likely to respond to the electronic survey, leading to undercoverage bias. This may explain the low proportion of participants with more than 20 years of clinical experience, with most participants having 1-20 years of experience. Nonrandom sampling and possible misinterpretations of the survey questions also pose drawbacks to the study design. These limitations may have influenced the study's findings, and the authors advise caution in generalizing the results to other regions. Future studies could benefit from more robust study designs, such as conducting in-person interviews, to address these issues.

Nevertheless, the strength of the study lies in its diverse participant pool, which includes general dental professionals and specialists from various dental fields. Additionally, the findings contribute to the limited literature on the topic in the context of Mumbai. The study emphasizes the importance of clinical experience and interdisciplinary efforts in the prevention and management of MRONJ.

## Conclusions

Despite the limitations of the present study, it was found that the majority of dental practitioners were familiar with the terminology of MRONJ. However, proper knowledge regarding the drugs that affect the disease and its clinical and radiographical presentations is needed. Awareness of treatment strategies and recent advances was notably low among general dentists and other dental specialists apart from OMFS. A lack of an interdisciplinary approach was also observed among the practitioners. There is a clear need for enhanced education and awareness on this subject, which can be achieved by conducting interdisciplinary surveys, conferences, and seminars. Integrating MRONJ education into dental curricula or organizing targeted workshops can help foster awareness and ensure proper management protocols. Premedication dental evaluation has been shown to significantly reduce the incidence from less than 10% to almost negligible levels. A need for a survey to assess awareness among medical physicians could highlight the importance of professional collaboration. Future research should focus on addressing the disease through these collaborative efforts.
